# 

**Published:** 1893-06

**Authors:** 


### Communicated.

### ILLINOIS STATE DENTAL SOCIETY.

The twenty-ninth annual meeting of the Illinois State Dental Society was held in conjunction with the Iowa State Dental Society at Rock Island and Davenport, May 9-12, 1893. The following officers were elected for the ensuing year:

Garrett Newkirk, Chicago, President; J. W. Cormany, Mt. Carroll, Vice-President; Louis Ottofy, Chicago, Secretary; W. A. Stevens, Chicago, Treasurer; F. H. McIntosh, Bloomington, Librarian.

LOUIS OTTOFY, *Secretary.*

---

### NORTHWESTERN UNIVERSITY DENTAL SCHOOL.

The graduation exercises of the Northwestern University Dental School were held on the afternoon of April 25, at Central Music Hall, Chicago.

Six students were graduated, as follows: Benjamin Merrill Ford, Murray Gordon Matteson, Jared Michael Garman, Philip Albert Pyper, Chas. Hazen Gale, Chas. Arthur Templeton.

E. NOYES, D. D. S., *Sec'y.*

### UNIVERSITY OF BUFFALO, DENTAL DEPARTMENT.

The first annual Commencement Exercises of the Dental Department of the University of Buffalo were held in connection with those of Medicine and Pharmacy, in Music Hall in the City of Buffalo on the evening of May 2nd, 1893.

The examinations before the Board of Curators, which comprises the Dental Examining Board of the State, lasted during the day. After these were finished, the Board held a meeting with doors closed to all, and after thoroughly canvassing their merits, unanimously recommended each of the candidates as well qualified to receive his degree, which was conferred upon him by the Chancellor in the evening.

The number of matriculates for the session was forty-six. The graduates were as follows, each having presented a senior ticket from some reputable institution before joining the class: William Jonathan Crawford, Ohio; T. DeForest Phillips, New York; Edward Harry Lamport, New York; William Charles Smith, California; Daniel Hubbard Squire, New York.

### THE MISSOURI STATE DENTAL ASSOCIATION.

The twenty-ninth annual meeting of the Missouri State Dental Association will be held at Excelsior Springs, Mo., July 11, 12, 13, 14 inclusive. All dentists are invited to attend, as the meeting promises to be of great value to the profession.

WM. CONRAD,
321 N. Grand Ave., St. Louis, Mo.

### To Readers and Correspondents.

Communications intended for publication, and exchange journals and papers, should be addressed to the Editor of The American Journal of Dental Science, No. 845 N. Eutaw St. Baltimore, Md. All letters relating to the business of the Journal, or containing cash remittances, to be directed to Messrs. Snowden & Cowman. No. 9 W. Fayette Street, Baltimore, Md. Articles for publication should be received by the Editor at least two weeks previous to the first of the month on which the number in which it is designed for them to appear, is issued.

☞ The American Journal of Dental Science will contain fifty pages of reading matter in each number, making a volume of about Six Hundred pages,

EXCLUSIVE OF ADVERTISEMENTS

